# Edible Coatings for Fish Preservation: Literature Data on Storage Temperature, Product Requirements, Antioxidant Activity, and Coating Performance—A Review

**DOI:** 10.3390/antiox13111417

**Published:** 2024-11-19

**Authors:** Mia Kurek, Petra Pišonić, Mario Ščetar, Tibor Janči, Iva Čanak, Sanja Vidaček Filipec, Nasreddine Benbettaieb, Frédéric Debeaufort, Kata Galić

**Affiliations:** 1 Laboratory for Food Packaging, Faculty of Food Technology and Biotechnology, University of Zagreb, Pierottijeva 6, 10000 Zagreb, Croatia; ppisonic@pbf.hr (P.P.); mscetar@pbf.hr (M.Š.); kgalic@pbf.hr (K.G.); 2 Laboratory for Meat and Fish Technology, Faculty of Food Technology and Biotechnology, University of Zagreb, Pierottijeva 6, 10000 Zagreb, Croatia; tjanci@pbf.hr (T.J.); svidacek@pbf.hr (S.V.F.); 3 Laboratory for General Microbiology and Food Microbiology, Faculty of Food Technology and Biotechnology, University of Zagreb, Pierottijeva 6, 10000 Zagreb, Croatia; icanak@pbf.hr; 4 Joint Unit PAM-PCAV (Physico-Chemistry of Food and Wine Laboratory), Université Bourgogne-Franche-Comté, Institut AgroDijon, INRAé, Université de Bourgogne, 1 Esplanade Erasme, 21000 Dijon, France; nasreddine.benbettaieb@iut-dijon.u-bourgogne.fr (N.B.); frederic.debeaufort@u-bourgogne.fr (F.D.); 5 Department of BioEngineering, Institute of Technology, University of Burgundy, 7 Blvd Docteur Petitjean, 210780 Dijon, France

**Keywords:** fish storage, antioxidants role, edible coatings, fish oxidation, cold vs. frozen

## Abstract

Fresh fish is among the most nutritive foodstuffs, but it is also the most perishable one. Therefore, huge efforts have been made to find the most suitable tools to deliver fish of the highest quality to exigent consumers. Scientific studies help the industry to exploit the newest findings to scale up emerging industrial technologies. In this review article, the focus is on the latest scientific findings on edible films used for fish coatings and storage. Since today’s packaging processing and economy are governed by sustainability, naturality underpins packaging science. The synthesis of edible coatings, their components, processing advantages, and disadvantages are outlined with respect to the preservation requirements for sensitive fish. The requirements of coating properties are underlined for specific scenarios distinguishing cold and freezing conditions. This review raises the importance of antioxidants and their role in fish storage and preservation. A summary of their impact on physical, chemical, microbiological, and sensory alterations upon application in real fish is given. Studies on their influence on product stability, including pro-oxidant activity and the prevention of the autolysis of fish muscle, are given. Examples of lipid oxidation and its inhibition by the antioxidants embedded in edible coatings are given together with the relationship to the development of off-odors and other unwanted impacts. This review selects the most significant and valuable work performed in the past decade in the field of edible coatings whose development is on the global rise and adheres to food waste and sustainable development goals 2 (zero hunger), 3 (good health and well-being), and 12 (responsible consumption and production).

## 1. Introduction

Increasing food waste presents a significant challenge for the food packaging industry, particularly in fisheries, aquaculture, and related supply chains, where 35% of harvested food is lost or wasted. Innovation is crucial to reduce these losses, as poor harvesting practices and spoilage contribute significantly to waste. Low-quality fish not only diminishes the nutritional value of meals but also reduces income for aquatic food workers. Even though edible coatings have long been used in the food industry, they still encounter many challenges on a commercial scale. However, these coatings remain a promising and innovative technique for fish preservation, effectively extending the shelf life of products. Beyond preservation, edible coatings and films improve the appearance, safety, convenience, and overall quality of food packaging while also reducing environmental impact.

**Figure 1:**
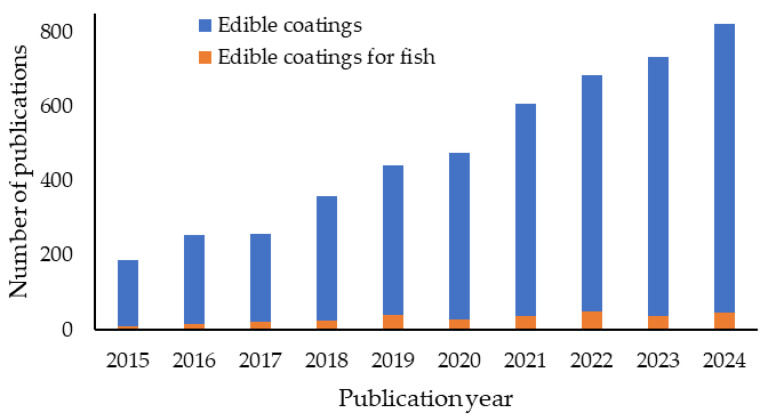
Paper publications on edible coatings or edible coatings for fish based on the Scopus database from 2015 to 2024 and the search term “edible coatings” or “edible coatings for fish”.

Furthermore, the use of natural and plant-based materials aligns with the increasing demand for sustainable and environmentally friendly packaging solutions. The scientific interest (Figure 1) in edible coatings for various foodstuffs, including fish and fishery products, has grown in the past decade due to promising results in enhancing product shelf life. In 2023, the global seafood packaging market size was valued at EUR 16 billion and is projected to grow to EUR 25 billion by 2032, dominated by the Asia Pacific region [1]. Globally, the market is mostly active in Northern America and Europe, specifically in the USA (e.g., MonoSol LLC., WikiCell Designs Inc., JRF Technology LLC., Safetraces, Inc.; BluWrap), and UK (e.g., Tate & Lyle Plc, Skipping Rocks Lab, Devro plc), followed by Australia and Asian countries. Characterizing coating formulations presents a challenge, both in a solution and once the coating has been applied, as it becomes an inseparable part of the product. Due to this, formulations are often spread onto foods, cast into self-standing films at the lab scale, or extruded on a commercial scale for easier analysis and testing.

Accordingly, various reviews are provided at different intervals, considering different aspects of application, and providing exhausting tables with listed film functions [2,3,4,5,6,7,8,9,10]. Despite this, there is no work that summarizes fish product storage and coating requirements. Understanding spoilage processes is crucial for developing effective coatings that extend the shelf life and maintain the quality of fresh fish. This review provides updated information on fish coatings tailored to the specific needs of the product during storage. Additionally, it addresses factors influencing the coating process, adhesivity challenges, and their impact on product characteristics.

## 2. Key Coating Components

### 2.1. Main Matrix Structure

The key components in coating design include food-grade polymers, solvents, and additives. Coatings are mainly made from polysaccharides, proteins, lipids, surfactants, plasticizers, bioactives, and fillers [4,11,12,13]. The primary purpose of the coating is to act as a protective layer that reduces gases and water vapor permeation, thereby lowering oxygen levels and microbial, enzyme, and oxidative reactions in the stored product [14]. Lately, smart edible films have been developed for the real-time monitoring of food freshness and successfully applied on various fish [15,16,17,18,19]. Although polysaccharide-based coatings such as chitosan provide a good barrier to O_2_ and CO_2_ and exhibit excellent mechanical properties, they present a poor barrier to water vapor permeation (WVP) due to their hydrophilic nature [20]. Proteins are highly effective barriers against gases and lipids, especially at low humidity levels. In contrast, lipids have limited mechanical strength but exceptional resistance to water and low surface energy because of their hydrophobic characteristics. Combining the best properties of each component allows designing composite material with optimal functionality.

The development of coatings made from food-grade biopolymers (food ingredients and/or food additives like film-forming agents or thickeners, etc.) such as polysaccharides (e.g., alginates, chitosan, pectin, and natural gums) and proteins (e.g., zein, whey protein concentrate, collagen, and gelatin) is increasing. Coatings should not change or change in a positive way the texture, flavor, and aspects of the fish product. However, the most important parameter to consider for the formulation of the coating is the main function targeted. This depends on the fish species and conditions of packaging and storage. For instance, for white fish fillets stored in frozen conditions in cardboard boxes without plastic film wrapping, the coating must be formulated to prevent moisture loss and surface dehydration. For pelagic fish, the target is to reduce fish fat oxidation, and therefore hydrocolloids with high oxygen barrier properties have to be selected. There is no clear literature data regarding the compatibility of the type of coating components in line with the type of fish to protect. It is clear that lipids will be focused on moisture transfer reduction, while considering the physical state of the lipids with respect to the temperature to prevent crack formation with cooling due to brittleness [21,22]. Dealing with oxidation, hydrocolloids receive the most focus but depend on the moisture content and water activity, which is only temperature-dependent below zero Celsius. Indeed, hydrocolloids’ performance against oxygen permeation highly depends on the moisture level, and oxygen permeability could vary by several orders of magnitude [22,23]. When coating may entrap bioactive compounds, the formulation should be focused on the ability of the coating to quickly release the active agents at the beginning of the packing step, followed by a slow release during storage. This has to be tuned according to interactions of the coating with the fish surface composition and considering the surrounding conditions (water activity, the state of water in contact with the coating, and temperature) [3,24]. The combination of both lipid and hydrocolloids as multilayer-based or emulsion-based structures is often the way to reach the target goals and monitor bioactivity [25,26].

Among the advantages of using hydrocolloids and lipids for fish applications, their availability at economically sustainable costs, i.e., being found in large amounts in various kinds of matrices in nature, is of key importance. Moreover, the wide range of hydrocolloid/lipid combinations with different structures and chemical functions that could interact with many hydrophobic or hydrophilic active compounds is considered in coating formulation.

### 2.2. Oily Compounds

To overcome sensibility to water, lipid compounds found in essential oils (EOs) or lipid fractions and waxes (like palm wax, carnauba wax, fatty acids, etc.) are added to the polysaccharide matrix [27,28]. Wang et al. [29] showed that chitosan with wampee seed essential oil decreased the WVP of film, and when used as a coating for golden pompano (*Trachinotus blochii*) fillets, it prevented the loss of moisture from fish flesh. Similarly, the incorporation of hydrophobic EOs into the hydrophilic chitosan polymer matrix has been found to improve its effect as a vapor permeability barrier [30]. For instance, pompano fillets coated with chitosan (2%) and clove essential oil (0.16%) had the lowest moisture loss (72.9%) at 120 days post-freezing compared with samples without modified coating [31].

Apart from being a source of lipid fraction, EOs and their compounds derived from spices and seeds (like cinnamon, clove, turmeric, etc.), leaves and flowers (tea, etc.), herbs (oregano, rosemary, basil, coriander, thyme, mint, etc.), and fruit and vegetable waste and byproducts (from garlic, fennel, orange, cucumber, grape, olive, etc.) are rich in secondary metabolites, such as phenols, flavones, terpenes, ketones, aldehydes, and alcohols [3,32,33,34,35,36]. Therefore, they provide antimicrobial and antioxidant activity to treated fish products [3,12,37,38,39,40]. The lower oxidation is likely due to the antioxidant’s presence in active films, and thus the coating effectively prevents the fish from being exposed to oxygen, thereby significantly slowing down the oxidation of fish fat [41]. Further elaboration on this is given in Section 3 and Section 4. However, when it comes to real fish application, the number of available studies rapidly decreases (only 118 in the Scopus database when searching for “essential oil fish preservation”). These papers are mostly dedicated to its major drawback, which is often attributed to the strong sensory impact of volatile compounds found in essential oils [42,43]. Therefore, choosing the right essential oil is crucial; it must be effective without compromising the quality of the fish. Still, due to the increase in consumers’ awareness about food safety and quality, the use of natural compounds, such as spices and herbs, remains of particular interest. Therefore, it is essential to inform consumers about the sensory alterations linked to preservative coatings [44,45]. The interaction of essential oils can be considered in two directions: (1) the interaction with a polymer matrix and the effect of EOs on its properties, and (2) the interaction with coated food products. Due to changes in the matrix structure, EOs can negatively impact mechanical properties, water sensitivity, and gas barrier performance. For instance, the presence of EO droplets may weaken the intermolecular interactions between polymer chains, acting as a plasticizer and ultimately increasing the ductility of the film. This effect can lead to greater elongation at break [46]. Interestingly, while some essential oils enhance film solubility, others appear to reduce it. For example, ref. [47] discovered that adding thyme essential oil microcapsules increased the solubility of starch films, which is attributed to the hydrophilic nature of the microcapsules. When considering gas barrier properties, hydrophobic compounds can facilitate the permeation of oxygen. As plasticizers, EOs may enable gas molecules to penetrate through oil/polymer interfaces, especially in films with higher concentrations of essential oils, creating channels for oxygen diffusion [48]. It is important not to overlook the sensory impact, which has often been noted in fish [42,43]. Enhanced communication plays a crucial role in addressing any potential consumer worries about the visual appeal of uncooked fish while also improving overall safety measures. Hao et al. [49] outlined different methods for applying EO treatment to chill-stored fish, offering insight into its impact on the efficacy of the most commonly used EOs (enhancement or suppression).

### 2.3. Solvents

When designing a coating, it is also important to carefully select the solvents used. For example, chitosan is a cationic polymer soluble in acidic media. Therefore, a decrease in tissue pH occurs immediately after coating, so the polymer itself can ultimately act as a biopreservative by enhancing the acidity of the fish muscle. However, it might also immediately change the sensory aspect of the tissue due to changes in texture and color. Even though it is preferable to keep the pH levels in fish tissue low, the choice of the solvent, as well as the pH of the final coating solution, must be made with precaution. In fact, a rise in pH suggests the presence of alkaline substances like ammonia that are produced by microbial activity during the spoilage of fish muscle [50].

### 2.4. Nanomaterials

Cetinkaya and Wijaya [51] outlined the good state of the art of nanomaterials designed to enhance the shelf life and quality of seafood products. Novel nanomaterials are currently being formulated in the shape of nanotubes (such as carbon and hallosyte) or as GRAS nanocarriers for intelligent monitoring and food preservation [52,53]. Additionally, nanogels (e.g., nanogels containing EOs or nano-oleogel composed of whey protein isolate and soy lecithin to mask undesirable fishy scents [54]), functionalized nanocrystals (e.g., carboxymethyl cellulose/cobalt-based metal–organic with UV-blocking, antibacterial, and cobalt-releasing properties for shrimp packaging [55]), solid lipid nanoparticles [56], nanofibers [57], and nano-fillers are also being developed. Nanoliposomes are generally stable and promote the controlled release of active compounds over time, thus upholding explicit application as a coating [58]. Novel research studies have demonstrated that nanoethosomes and nanophytosomes offer a promising alternative to nanoemulsions by ensuring extended sustained release and targeted delivery. Nanoemulsions are colloidal systems composed of two or more liquids (water and oil). The oil/water types are preferable nanoemulsions for lipophilic bioactive compounds, and water/oil/water are preferable for hydrophilic ones [52]. Additionally, nanoparticles incorporated into ice, nanoprecipitates, 3D nanonetworks, and nanosheets as the latest innovative concepts have been established, although further exploration of these systems is still needed.

While nanotechnology in food packaging offers promising benefits, especially in terms of food preservation and safety, there are still concerns about the migration of nanoparticles and their potential toxicity. Regulatory bodies are working to ensure that any nanomaterials used are safe, but continued research is essential to fully understand the long-term implications. Different countries have different regulatory frameworks. The European Food Safety Authority (EFSA) has specific guidelines for evaluating the safety of engineered nanomaterials in food packaging. More in-depth studies are needed to understand how different nanomaterials interact with the human body and the environment. Scientists are looking into creating nanomaterials that are more biodegradable and pose fewer risks to human health and ecosystems. By creating packaging that reacts to environmental changes (e.g., temperature, humidity), nanotechnology can alert consumers to food spoilage or contamination, reducing the risk of consuming unsafe food.

## 3. Improper Storage Conditioning and Spoilage Mechanism

**Figure 2:**
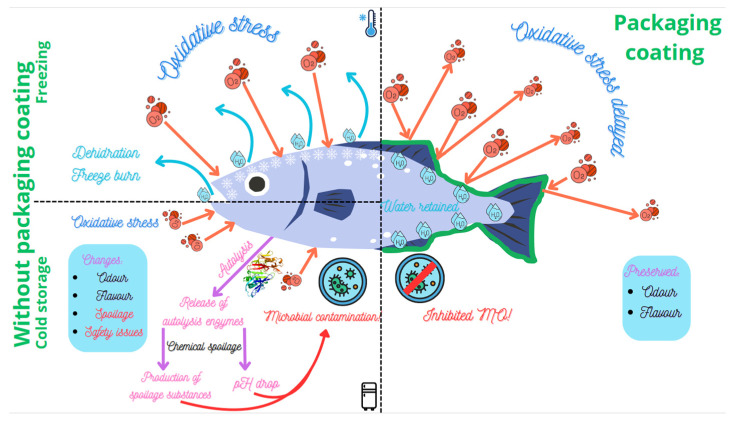
Illustration of fish spoilage and changes upon storage in cold or frozen, without and with edible coating.

Fresh or minimally preserved fish can experience a decline in quality due to microbial decomposition. This spoilage results in a 25–30% loss of marketable fish [59,60,61]. Fish flesh creates an ideal environment for microbial and biochemical spoilage: high moisture content (70–80%), an abundance of small molecules, and neutral pH. This ultimately leads to the rapid deterioration of sensory and nutritional attributes [11]. A schematic presentation of changes in relation to storage is given in Figure 2.

The loss of freshness of the fish after capture and its subsequent spoilage of the fish are the result of three basic mechanisms: enzymatic autolysis, oxidation, and microbial growth. The numerous reactions overlap in the time from capture to spoilage, resulting in a gradual loss of freshness/quality, leading to spoilage and shelf life end. Although the mechanisms leading to a loss of quality in fresh fish are quite complex and influenced by a larger number of factors, this process follows a characteristic pattern: a loss of freshness during the first stages of storage is mainly due to autolytic spoilage mechanisms, followed by microbial degradation leading to spoilage until the fish is no longer fit for consumption [62].

The sensory quality of fresh fish during storage is assessed on the basis of various attributes such as appearance, odor, and texture, which reflect its freshness and overall quality. In European Union (EU) legislation, the sensory quality of fresh fish is assessed using a classification system that categorizes fish into three different levels of freshness: E (Extra), A, and B. Fish that do not meet the criteria of category B are considered unfit for human consumption (EC) No. 2406/96.

### 3.1. Autolytic Enzymatic Spoilage

Enzymatic autolysis begins immediately after the death of the fish with the switch from an aerobic to an anaerobic mechanism of adenosine triphosphate (ATP) formation and the accumulation of lactic acid, which leads to a drop in the pH of the fish tissue. ATP is gradually degraded through a series of reactions that lead to the accumulation of metabolites such as hypoxanthine. The depletion of ATP reserves leads to the onset of rigor mortis, i.e., the stiffening of muscle tissue due to the binding of the myofibrillar proteins actin and myosin [63]. Later stages of autolytic spoilage are characterized by the activity of endogenous proteases and lipases, which lead to the degradation of proteins and the gradual resolution of rigor mortis, the breakdown of lipids, and the creation of an environment favoring microbial growth [64]. The key enzymes responsible for autolytic degradation, including chymotrypsin, cathepsins, trypsin, and lipase, are located in different parts of the fish, such as the hepatopancreas and stomach [65]. Proteolysis leads to the formation of free amino acids and peptides, which not only deteriorate the texture of the fish but also provide nutrients for the growth of microorganisms, which in turn contribute to spoilage.

In addition to enzymatic degradation, the formation of compounds such as trimethylamine (TMA) and formaldehyde can also contribute to spoilage. TMA is formed by the enzymatic reduction of trimethylamine N-oxide (TMAO) and leads to undesirable odors. This process, which is mediated by TMAO reductase, is particularly common in fish species in which TMAO is an important component [61]. Formaldehyde produced from TMAO crosslinks proteins, leading to the hardening of fish muscle and reduced water holding capacity. The hydrolysis of triglycerides by lipases releases free fatty acids, which tend to oxidize and contribute to the formation of characteristic off-odors associated with spoilage [66].

### 3.2. Microbial Spoilage

Fish is an excellent medium for microbial growth due to its high water content, relatively high pH, and abundance of nutrients such as proteins and non-protein nitrogen compounds typically found in fish tissues. Microbial proliferation leads to changes in odor, appearance, taste, and texture. The composition of the initial microflora depends largely on the water environment, with bacterial species such as *Pseudomonas*, *Alcaligenes*, *Vibrio*, *Serratiam*, and *Micrococcus* commonly found on freshly caught fish [67]. When these microorganisms proliferate, they metabolize the non-protein nitrogenous compounds present in fish, producing organic acids, alcohols, sulfides, and aldehydes that contribute to the characteristic off-flavors and odors of spoiled fish, as well as biogenic amines such as histamine, cadaverine, and putrescine, which are a health and food safety concern [68,69,70]. In addition, proteolytic, lipolytic, and nucleolytic bacterial enzymes can degrade proteins, lipids, and nucleotides and alter the texture and flavor of fish, leading to spoilage [61].

Specific spoilage bacteria, including *Pseudomonas* spp., *Shewanella putrefaciens*, *Photobacterium phosphoreum*, and different *Vibrionaceae*, are mainly responsible for spoilage in chilled fish [71,72]. These bacteria thrive and use trimethylamine oxide (TMAO) as an energy source in addition to free amino acids and other small peptides, resulting in the production of volatile compounds such as trimethylamine (TMA), which is responsible for the fishy odor often associated with spoiled fish [73].

Pathogenic bacteria will overgrow chilled fish and cause final deterioration where fish is no longer organoleptically, visually, and microbiologically acceptable for consumption.

In addition, both Gram-negative and Gram-positive bacteria can contribute to spoilage under certain conditions. While Gram-negative species predominate in fresh and chilled fish, prolonged storage or certain processing conditions can favor the growth of Gram-positive bacteria such as *Micrococcus*, *Corynebacterium*, *Bacillus*, and *Brochothrix thermosphacta* [74]. The presence of these spoilage bacteria leads to textural changes, off-flavors, and the production of toxic compounds such as histamine, which is particularly stable and resistant to common food preservation methods such as cooking or freezing [65,75].

### 3.3. Oxidative Spoilage

Lipid oxidation is another factor that contributes to fish spoilage and degradation, especially due to the high content of polyunsaturated fatty acids (PUFAs) in seafood [65]. This process leads not only to the development of off-flavors and odors but also to a loss of nutritional value, including the loss of fat-soluble vitamins and unsaturated fatty acids [76]. Autooxidation and enzymatic oxidation are two distinct mechanisms of lipid oxidation in fish. Autooxidation is a free radical-mediated process that occurs spontaneously in the presence of oxygen. It involves three stages: initiation (the formation of free radicals), propagation (a reaction with oxygen to form peroxyl radicals), and termination (when radicals combine). A recent review paper showed that lipid oxidation in fish could be due to decreased primary and secondary lipid oxidation products in red drum or by preventing oxygen diffusion via the alginate layer in rainbow trout [77]. Furthermore, a reduction in lipid oxidation can be due to the absorption or scavenging of undesirable compounds such as free radicals. Free radicals generated by heat, the presence of metals, and light react with unsaturated fatty acids to form hydroperoxides and other secondary oxidation (aldehydes and ketones). Enzymatic oxidation, on the other hand, occurs via enzymatic activity, primarily by lipoxygenases, which oxidize fatty acids directly. Triacylglycerols and phospholipids in fish muscle are first hydrolyzed by lipases and phospholipases, increasing the content of free fatty acids, which are susceptible to oxidation.

In fish muscle, the oxidation process is exacerbated by the presence of pro-oxidants such as hemoglobin and myoglobin [78]. Bleeding the fish can reduce the rate of oxidation by removing these pro-oxidants, thereby mitigating spoilage [79]. Secondary products of lipid oxidation not only cause a rancid taste but also interact with proteins, leading to denaturation and a loss of functionality, further reducing fish quality [79].

The oxidation of lipids can occur via both enzymatic (endogenous or microbial) and non-enzymatic pathways, involving reactions between oxygen and the double bonds in fatty acids, which are abundant in fish. This leads to a significant deterioration of sensory properties, such as odor, color, and texture, as well as nutrient losses. In addition, oxidation products can promote the denaturation of proteins and the degradation of endogenous antioxidant systems, accelerating spoilage [76].

By providing a functional barrier, edible coatings minimize the risk of these oxidative reactions. A barrier can be achieved by polymers with coatings that have a low permeability to gases, like chitosan, or by modification with the addition of functional additives and antioxidants by crosslinking reactions, among other options.

The spoilage process and the prevailing spoilage mechanisms are largely influenced by the storage temperature (freezing vs. chilling). Lowering the temperature significantly reduces the rate of enzymatic, chemical, and microbial processes and extends the shelf life. For chilled fisheries stored at the temperature of melting ice, all three mechanisms contribute to spoilage, but microbial spoilage has a decisive effect on their acceptability. This is because the number of microbes can reach high numbers in later stages of storage, leading to the degradation of fish muscle components and the production of many compounds that affect the odor and taste of the fish more than, for example, the products of oxidation processes. On the other hand, microbial activity is completely stopped in frozen products, and quality deterioration is mainly the result of oxidative reactions leading to rancidity. In addition, the quality of frozen products can be affected by surface dehydration and freezer burn during prolonged storage, resulting in color and texture changes (Figure 2). Therefore, the storage conditions and the properties of the packaging material must be taken into account when selecting the packaging for each application.

## 4. Cold Storage

High-quality fresh seafood is in global demand, but due to its perishable nature, it requires meticulous handling, especially during cold storage. The primary concerns during storage are preventing microbial growth and the oxidation of proteins and unsaturated fatty acids, both of which contribute to a loss in nutritional value and the development of unpleasant odors. Various preservation techniques can help manage these challenges. Oxidation can be minimized using films that present a strong barrier to oxygen or via the inclusion of antioxidants. Antioxidants stabilize polyunsaturated fatty acids, slowing down processes like rancidity and discoloration by chelating metals and scavenging singlet oxygen. This, in turn, helps maintain the nutritional value of the fish [61,80].

### 4.1. Antioxidant Engagement

Oxidation and microbial spoilage are the primary mechanisms responsible for the deterioration of fresh fish during cold storage. Coatings with antioxidants help preserve or improve fish quality by slowing these processes.

Antioxidants are substances that protect cells from oxidative damage through various mechanisms [81]. They can scavenge reactive species that initiate peroxidation, break auto-oxidative chain reactions triggered by reactive oxygen species, chelate metal ions to prevent the formation of reactive oxygen species, or decompose peroxides and reduce localized oxygen concentrations. Most natural antioxidants can be categorized into three distinct groups: phenolic compounds, vitamins, and carotenoids. In addition to being the primary components responsible for antioxidant activity, they frequently exhibit antibacterial and antifungal properties.

Antioxidants are classified as primary or secondary, depending on their action mechanism. Some exhibit multiple mechanisms and are referred to as multi-function antioxidants [3,82]. They can also be categorized as natural or synthetic, with natural antioxidants being increasingly preferred. Synthetic antioxidants that have received approval for use in foods include butylated hydroxytoluene (BHT), butylated hydroxyanisole (BHA), propyl gallate (PG), octyl gallate (OG), dodecyl gallate (DG), ethoxyquin, ascorbyl palmitate (AP), and tertiary butylhydroquinone (TBHQ). Preference for natural antioxidants is driven by factors such as heightened health awareness among consumers and a growing acceptance of green materials. Most natural antioxidants are derived from plants, vegetables, herbs, or spices and include phenolic compounds, such as phenolic acids (like caffeic and p-Coumaric), coumarins, flavonoids, tannins, essential oil compounds and volatile phenols (thymol, carvacrol, eugenol, cinnamaldehyde, etc.), phospholipids, tocopherols (α, β, γ, or δ), carotenoids (β-carotene, β-cryptoxanthin, and lycopene) and organic acid such as ascorbic and citric acids [83]. Additionally, some polymers, such as chitosan, fucoidan, polylactones, and lignin, also possess antioxidant properties [84,85].

Antioxidant activity can be achieved via different mechanisms, such as singlet oxygen deactivation, peroxide enzyme inhibition, the chelation of transition metals, enzymatic detoxification of reactive oxygen species, and their stabilization through hydrogen radical transfer.

Edible polymers in these coatings act as reservoirs for bioactive compounds, allowing for their slow, controlled release onto the fish surface. Due to food safety and health concerns, there is a strong push to replace synthetic antioxidants with natural alternatives.

The controversy arises because the ability to exhibit antioxidant and pro-oxidant behavior depends on various factors [86]. Under aerobic conditions, they can generate superoxide radicals and dismutate to H_2_O_2,_ which forms reactive oxygen species. Carotenoids and flavonoids, often regarded as antioxidants, can exhibit pro-oxidant effects at high concentrations due to autoxidation. However, each compound responds differently to environmental conditions. In fish tissue, the presence and activation of heme proteins as natural pro-oxidants play a critical role in initiating oxidation. Hence, overload in antioxidants is not a good solution since it might lead to the opposite effect. The type of fish as reacting media must also be considered since tissue richer in iron can be sensitive to hemoglobin-mediated lipid oxidation [87].

However, measuring antioxidant activity directly from the coating can be challenging, as it becomes an integral part of the product once applied. In such cases, the antioxidant potential is typically evaluated by monitoring the oxidation levels in the fish itself rather than the coating. This method provides an indirect assessment of the coating’s effectiveness in preventing spoilage.

The antioxidant capacity has been extensively studied [88,89,90,91]. Different methods have been suggested. However, oxidation levels, pH, solvent, and other reaction parameters may affect an antioxidant’s quantitative in vitro capacity [92]. The evaluation of a substance’s capacity to scavenge free radicals yields a quantitative value for the overall levels of antioxidants in biological samples, primarily plants and spices, which are incredibly complex systems. This process does not evaluate the antioxidant levels of individual substances because it would be time-consuming. The most common tests include the detection of electron or radical scavenging, known as the DPPH assay (2,2-diphenyl-1-picrylhydrazyl), the ABTS assay (2,2′-azinobis3-ethylbenzothiazoline-6-sulfonic acid), the FRAP assay (ferric-reducing antioxidant power), and β-carotene bleaching assay. The DPPH scavenging ability is an indicator of radical scavenging, whilst the inhibition ratio in the β-carotene bleaching assay reflects the ability to inhibit oil oxidation. Direct measuring in a coating formulation first requires isolation (extraction) of the antioxidant from the matrix (coating or film, pieces of known composition and mass), which is generally performed by an adequate solvent.

**Figure 3:**
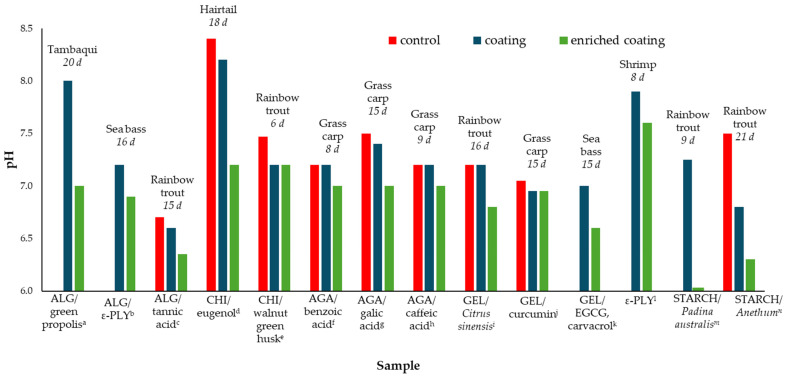
pH values of fish samples with antioxidant active coatings during cold storage. ALG—alginate, CHI—chitosan, AGA—agarose, GEL—gelatin, ε-PLY—ε-polylysine, EGCG—epigallocatechin gallate. ^e^ [94]; ^l^ [100]; ^c^ [101]; ^m^ [102]; ^g^ [103]; ^f^ [104]; ^k^ [109]; ^j^ [110]; ^a^ [111]; ^b^ [112]; ^d^ [113]; ^h^ [114]; ^i^ [115]; ^n^ [116].

During cold storage, the pH tends to decrease within the first few days [93,94]. This was observed for *Sardina pilchardus* [95], *Ctenopharyngodon idella* [96,97,98], *Dicentrarchus labrax* [99], *Litopenaeus setiferus* [100], *Oncorhynchus mykiss* [101,102,103,104], and *Sparus aurata* [105,106]. The improper storage usually leads to autolysis, which causes tissue softening, belly bursting, and the production of hypoxanthine and lactic acid produced by muscle glycolysis in postmortem fish that change the pH. Since fish are extremely sensitive to microbial proliferation, in the later storage stages, due to microbial activity, the pH might increase. This increase is attributed to the accumulation of nitrogenous compounds, such as ammonia, amino acids, trimethylamine, hydrogen sulfide, indole, and other volatile bases, caused by the growth of microbial and endogenous enzymes [107]. Edible coatings may have a crucial role in protecting against substrate decomposition and the growth of microorganisms, causing a nearly constant pH behavior during storage [108]. Since the addition of antioxidants further improves microbial stability, it is to be expected that formulations with antioxidants would contribute even more to the pH stability (Figure 3). Song et al. (2023) [109] showed that there was a strong influence of antioxidants and correlation between the values of DPPH⋅ scavenging activity and ABTS+ scavenging and pH. As the antioxidant activity of gelatin enriched with epigallocatechin gallate increased, the pH decreased. Therefore, for samples with DPPH values of 1.2, 36.90, and 92.60%, pH decreased from 6.81 to 6.72 and 6.68, respectively. However, the presence of excessive hydrophobic additives reduced the compactness of the coatings, which weakened the water barrier properties of the coatings and caused greater weight loss, as was shown for gelatin coatings enriched with curcumin/βcyclodextrin [110].

Fish also suffer from a rapid loss of hardness during storage, which extremely impacts their short shelf life. Coatings protect fish muscle from softening to a lesser extent, like in the case of alginate/polylysine when used on seabass [112]. Softening can occur due to the degradation of fish protein by endogenous autolytic enzymes and susceptibility to microbial contamination. According to the literature [101,116,117], coatings enriched in antioxidants (e.g., EOs, tannic, quebracho tannin, and tannic and gallic acid) could contribute to maintaining a firmer texture. However, the storage duration must not be neglected since, due to tissue degradation, texture parameter hardness, cohesiveness gumminess, chewiness, and resilience tend to decrease [101].

**Figure 4:**
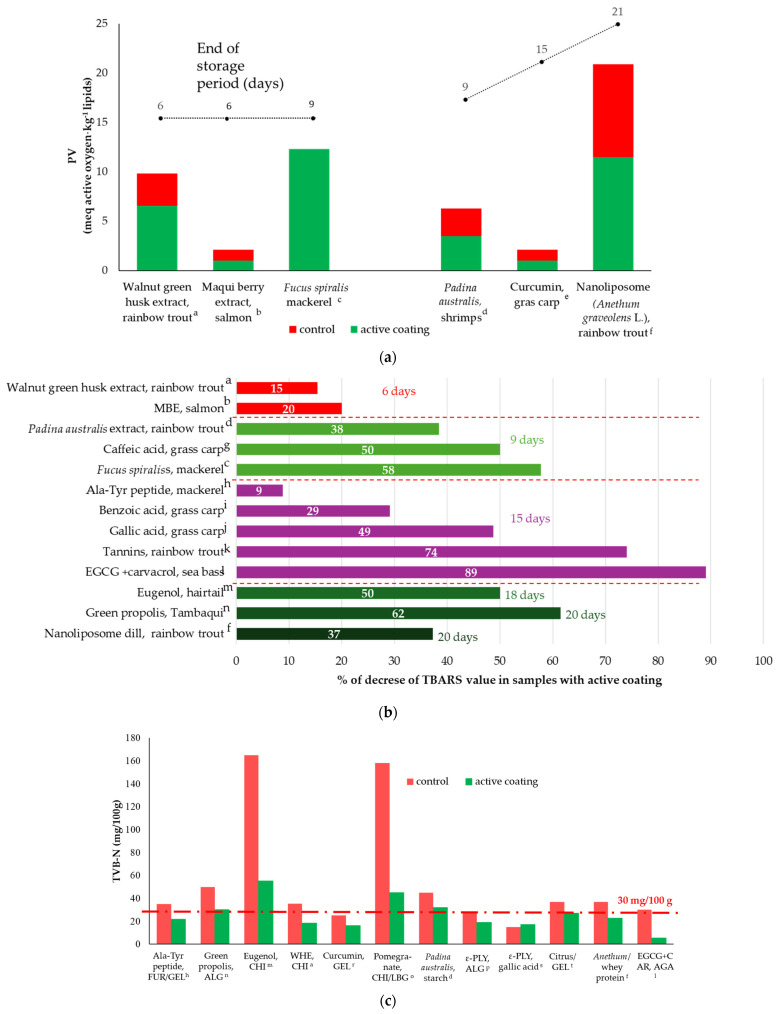
Evolution of (**a**) peroxide value (PV) and (**b**) reduction percentage in TBARS (Thiobarbituric acid reactive substances) and (**c**) TVB-N (Total volatile basic nitrogen) values in some fish samples with active antioxidant coatings during cold storage. [94]—a; [101]—k; [102]—d; [103]—j; [104]—i; [109]—l; [110]—e,r; [111]—n; [112]—p; [113]—m; [114]—g; [115]—t; [116]—f, [118]—b; [119]—c; [120]—h; [121]—o; [122]—s.

What makes a difference is that in cold storage, edible layers can form a compact film on the surface of the fish, which can effectively prevent the entry of outside air, especially oxygen. For this reason, the synergistic action with antioxidants is more than evident and described indirectly through lower peroxide, TVB-N, and TBARS values (Figure 4).

The peroxide value reflects the extent of primary lipid oxidation [123]. TVB-N is generally studied as a deterioration indicator, which detects the compounds containing ammonia and primary, secondary, and tertiary amines. The higher the TVB-N value, the more severe the corruption of the fish. The accumulation of biogenic amines in fresh fish has been mainly attributed to the growth of bacteria possessing amino acid decarboxylase activity, which is facilitated by a lack of hygienic conditions and strict temperature control during their storage [124]. Therefore, a high TVB-N value is not what people expect. Generally, TVB-N values show an upward trend with increasing storage time, but this can be slower in samples with antioxidants (AOs) (Figure 4b). The literature offers studies performed at different time intervals; however, the tendency seems to be the same but with a different extent depending on the type and initial antioxidant capacity of the active compound used, the polymer structure, and the type of fish and its lipid profile, and the storage often varies from 6 to 21 days.

The thiobarbituric acid (TBA) test measures malonaldehyde (MDA) produced due to the oxidation of fatty acids with three or more double bonds, and it measures other TBA-reactive substances such as 2-alkenals and 2,4-alkadienals. Fish flesh with off-flavor and taste should be rejected when the TBA value is beyond 2 mg of MDA/kg. A significant difference in TBA content was observed when using fish coating, and it was even more amplified after the addition of AOs (Figure 4c). Even though antioxidants may be applied in free form to fish, their potential is often improved when they are encapsulated in various polymeric systems [125].

**Figure 5:**
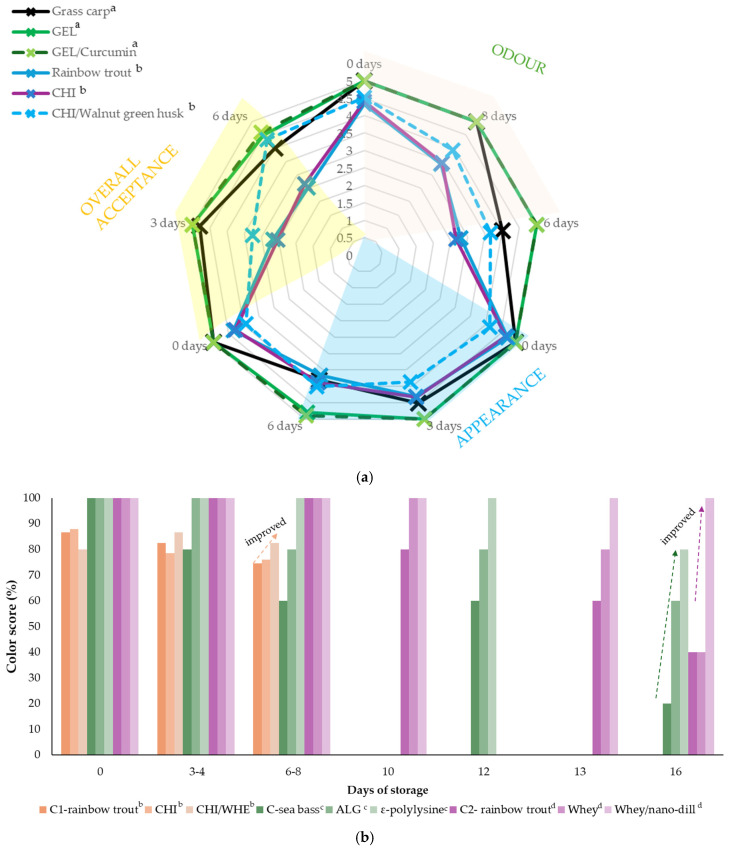

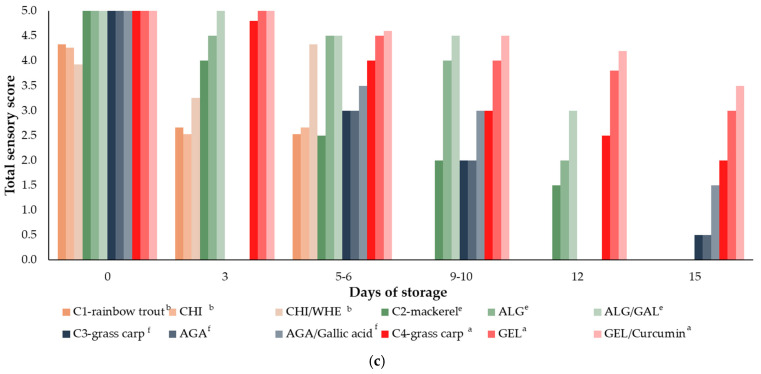
Changes in some sensory parameters and total sensory score in different fish samples as influenced by edible coatings and antioxidant compounds: (**a**) grass carp and rainbow trout in gelatin/curcumin and chitosan/walnut green husk-based coatings, respectively, for 6 days of storage. (**b**) color score (recalculated from the original data as score/max score) and (**c**) total sensory score (data adapted from ^b^ [94]; ^f^ [103] ^a^ [110]; ^c^ [112]; ^d^ [116]; ^e^ [117]). C1, C2, C3, and C4—control samples from each study.

Sensory evaluation is an intuitive way to characterize the quality of fish fillets during storage. Sensory rejection is generally caused by discoloration, physical changes, textural changes, slime or gas formation, or the development of off-flavors and off-odors. It was previously shown that the sensory scores change as the fish is being spoiled. However, there is clear scientific evidence that edible coatings improve sensory characteristics due to the inhibition of the degradation of various parameters, as described above. Even though it is believed that the addition of EOs might significantly impact the sensory quality, there are still publications indicating that the addition of antioxidants of this kind could indeed help to preserve fish color, odor, and taste. The literature data are summarized in Figure 5.

The development of new products is a crucial competitive indicator for companies operating in well-established markets. Adapting products to meet market demands helps differentiate them from their competitors. Today, consumers increasingly seek fish products that are ready to eat, safe, and meet specific claims that address their needs while maintaining desirable sensory qualities. Several factors influence consumers’ intent to purchase new food products, including intrinsic sensory attributes like appearance, taste, flavor, and texture, as well as various physiological and psychological aspects. Appearance plays a crucial role in consumers’ purchase intentions and acceptance, especially concerning fish products. To enhance this intrinsic factor, it is essential to develop strategies aimed at improving appearance. One potential solution is the use of edible coatings, which can prevent the formation of juices in fish packages placed in retail plastic trays. These juices are often unappealing to consumers, and by addressing this issue, the product can become more acceptable. Based on the reviewed articles in this chapter, it can be concluded that samples whose sensory attributes were not significantly impacted may be considered for further use. Nevertheless, there is a risk of food neophobia, particularly due to insufficient evidence regarding the safety and efficacy of coatings for consumer use. Proper education and labeling, in accordance with Codex Alimentarius and European Commission regulation 2004/1935/EC, which endorses the concept of active packaging with the intentional release of active agents, could lead to a greater acceptance rate. Currently, no surveys have been conducted on consumers’ attitudes toward the use of edible coatings for fish. This lack of research is partly due to the limited availability of marketable examples. In contrast, well-known fruit coatings have shown a gradual acceptance among consumers. When combined with refrigerated storage, these coatings enhance both the safety and quality of food products, as they are made from natural ingredients [126].

### 4.2. Inhibition of Microbial Growth

**Figure 6:**
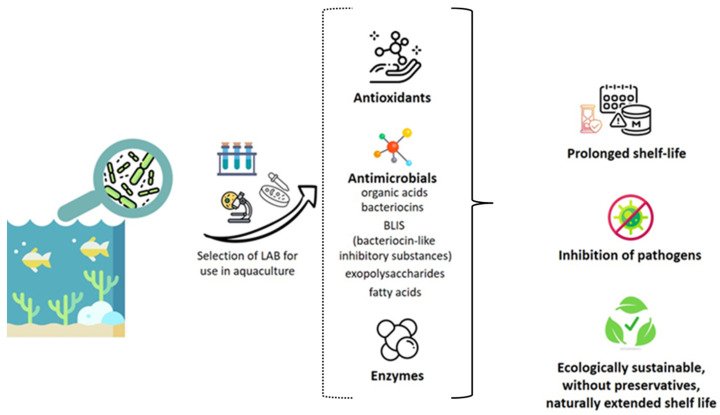
Use of marine origin lactic acid bacteria in aquaculture.

In the case of aquaculture products, there is an increase in filleted and prepared fish and shellfish. Consumers want more natural food products, and consequently, the demand for preservatives of biological origin rises. Therefore, biopreservatives, such as lactic acid bacteria (LAB) and/or their metabolites, can play an important role. LAB, such as *Lactiplanitbacillus* and *Lactococcus* show promise in inhibiting the growth of spoilage bacteria such as *Listeria monocytogenes* and *Pseudomonas* sp. without interfering with the quality of the product. LAB strains are crucial in the growth control of many food-spoilage and pathogenic bacteria by producing a variety of antimicrobial substances like acids, hydrogen peroxide, diacetyl, acetoin, and small peptide molecules known as bacteriocins, as well as competing for specific nutrients [127] (Figure 6). Since lactic acid bacteria have GRAS (Generally Regarded as Safe) and QPS (Qualified Presumption of Safety) status designated by the FDA and EFSA, among all other benefits, they are also safe to use. Certainly, the most interesting compound is nisin, a polypeptide tested in different food matrices, the only one approved by the FDA and listed under the European number E234 as a food additive [128,129].

Numerous research studies have been conducted concerning the biopreservation of aquaculture products using LAB, but they are mainly focused on shrimp, salmon, and trout [55,130,131,132,133], and a smaller portion of the research is based on marine fish. Meanwhile, research dealing with edible coatings of fish and shellfish is just beginning and is mainly based on a combination of nisin and probiotic strains [134,135].

LAB produce numerous metabolites, such as acids, bacteriocins, hydrogen peroxide, diacetyl, acetaldehyde, and acetoin, that exert antimicrobial activity toward pathogenic microorganisms. Therefore, LAB have a protective role and can be used as a preservative in different kinds of food, including aquaculture products, and extend the shelf life naturally without adding chemical agents [136,137].

Lopez de Lacey et al. [138] investigated the effect of the addition of agar films with green tea extract and two LAB strains—*Lacticaseibacillus paracasei* L26 and *Bifidobacterium lactis* B94—to hake fillets. The results showed a reduction in total viable bacteria throughout the storage period as well as a decrease in total volatile basic nitrogen (TVB-N), trimethylamine nitrogen (TMA-N), and pH.

Wang et al. [139] discovered that edible coatings made from tartary buckweat pollysaccharide and nisin maintained quality and enhanced the shelf life of tilapia (*Oreochromis niloticus*) fillets during storage at 4 °C.

One new research study dealt with the extension of fresh trout fillet quality by combining carboxymethylcellulose and sodium caseinate with different LAB strains. These films reduced the number of viable bacteria and successfully extended the shelf life of trout fillets stored at 4 °C [140].

In line with the mentioned results, fresh fish is widely consumed and ranks among the most traded food commodities globally. Traditional preservation methods, such as vacuum and modified atmosphere packaging, can be expensive due to their high capital requirements, so research has focused on developing antimicrobial packaging systems as a cost-effective alternative. Available scientific data indicate that edible films and coatings enriched with various active agents can indeed inhibit microbial growth and slow nutrient degradation in fresh fish. This approach has successfully extended the shelf life of fresh fish fillets up 2 weeks, depending on the fish species, as summarized in Table 1, including rainbow trout, silver carp, grass carp, salmon, Japanese sea bass, red drum, golden pomfret, and hake. Antimicrobial coatings are generally made from gelatin, chitosan, chitosan–gelatin, gelatin–alginate, carrageenan, quince seed mucilage, whey protein concentrate, and whey protein isolate, all enhanced with various active agents. These include essential oils (EOs) from clove, cinnamon, oregano, thyme, and lemon, as well as glycerol monolaurate, α-tocopherol, lactoperoxidase, citric acid, licorice extract, grape seed extract, and tea polyphenols. Their antimicrobial effectiveness was assessed in situ against spoilage and pathogenic microorganisms. The results showed varying levels of effectiveness influenced by the active agent, its concentration, storage temperature, atmospheric composition (normal or modified), and the specific microorganism targeted.

Utilizing these formulations could significantly reduce fish waste and minimize economic losses for traders and retailers and consequently trends toward sustainability goals. Therefore, the industrial production and commercialization of these antimicrobial packaging solutions represent a promising opportunity for the packaging industry.

## 5. Frozen Storage

Freezing fish involves extracting heat from the fish body to decrease its temperature, typically ranging from −18 to −30 °C. This method has been widely employed to maintain fish quality as it inhibits biological and chemical reactions; slows the growth of microorganisms; and minimizes physical deterioration such as enzymatic activity, color alteration, and lipid oxidation [153].

Freezing is based on the formation of ice crystals, which grow larger in a slow process. This leads to the denaturation of proteins, the rupture of cell membranes, and the loss of water during thawing. If the freezing process is carried out quickly (in blast freezers, plate freezers, or cryogenic freezers), the crystals are smaller, resulting in a high-quality product. The quality of frozen fish depends on the initial quality of the fish before freezing, the freezing rate, the storage temperature, oxygen, and temperature fluctuations during storage. During freezing and frozen storage, the fish muscle undergoes changes in proteins (denaturation and oxidation) and lipids (oxidation), resulting in water loss during thawing, an increase in pH, changes in texture, and the development of rancidity. A fast-freezing speed, a low storage temperature without fluctuations, a suitable glaze, and packaging (vacuum and modified atmosphere, as in these cases, oxygen is removed from the pack while packaging material with adequate permeability ensures desired resistance against oxygen and moisture) result in a high-quality product.

Poorly packaged frozen fish may experience dehydration, surface drying, and oxidation. Thus, the primary objective of an edible coating during frozen storage is to offer sufficient barriers against water vapor and oxygen influence while preserving the desired pH level.

### 5.1. Prevention of the Dehydration

Dehydration, commonly referred to as freezer burn, occurs when fish tissue is exposed to extremely cold temperatures and temperature fluctuations. This issue can significantly affect the quality and nutritional value of the fish. This process alters ice crystal formation, as well as the structure of proteins and lipids. Water movement within the fish can impact its texture, while ice sublimation can affect its sensory properties, including taste and appearance. Additionally, surface dehydration from ice sublimation increases the fish’s exposure to oxygen, leading to the rapid oxidation of fats and oils, which results in rancidity, unpleasant flavors, and discoloration. Protein denaturation and microbiological growth can also occur. To mitigate these effects, it is crucial to select an edible coating for frozen fish that effectively binds water and creates a protective barrier. This barrier helps maintain the fish’s texture and prevent protein degradation by reducing moisture loss and limiting exposure to air. This is because the coating’s water retention capacity is believed to play a key role in preserving the myofibrils and their ability to retain water [154,155]. The coating performance during freezing is influenced by the stability of the coating crating molecules at low temperatures. Temperatures below zero could impact the crystallinity, structure, and mechanical properties of biological molecules used for coating production, especially when lipids, waxes, or oils are applied as emulsion systems since these can be destabilized during drying. A small increase in WVP, possibly due to structure imperfections, could develop because of fluctuating storage temperatures and an accompanying contraction and expansion of the lipids. However, this was not observed for frozen whey coatings [156]. To our knowledge, few studies have examined the effect of freezing on the physico-chemical, mechanical, structure, and barrier properties of food coatings. However, it was shown that freezing treatment is a facile and effective way to improve hydrogels’ mechanical properties but is strongly dependent on the gel’s moisture content [157,158].

For example, an increased amount of immobilized water was confined within the myofibrillar protein network when sodium alginate coating was used on yellow croaker (*Larimichthys polyactis*) [159]. The addition of hydrophobic essential oils to the hydrophilic polymer matrix has been shown to enhance its performance as a vapor barrier. Specifically, fish fillets coated with a mixture of 2% chitosan and 0.16% clove essential oil experienced the least amount of moisture loss (2%) after 120 days of freezing, outperforming other treatment groups (e.g., control 5%). Therefore, the blend of 2% chitosan and 0.16% clove essential oil could be a promising coating option for frozen fish fillets [31]. Izadi et al. [30] indicate that the addition of the shallot oil to the tomato seed mucilage coating might have increased the coating hydrophobicity, being the reason for the reduced loss of moisture in fish fillets measured as weight loss (3.5% or less) compared to the control treatment (6%). In a very recent study, it was shown that the use of seaweed extract-based films could be effective in preventing color changes in salmon exposed to freezing conditions and, consequently, reducing the appearance of freezer burn [160]. In addition, textural changes of fish flesh commonly appearing after defrosting were minimized in coated samples.

The optimal biopolymer network structure can efficiently inhibit dehydration in frozen fish samples by slowing down the diffusion rate of water molecules through the coating film or providing an extra water reservoir at the fillet surface (glazing), ultimately reducing freezer burn. As demonstrated earlier, the use of chitosan/gelatin coating resulted in enhanced moisture stability in freshwater carp fillets over a period of 4 months when compared to samples without coating [161]. Augusto et al. [160] showed that alginate-based films containing *Grateloupia* and *Sargassum* extracts provided improved protection for frozen salmon against freezer burn. This was attributed to the lipid-derivative compounds present in the *Grateloupia* extract.

Moreover, the color of fish is determined by the protein–water binding characteristics. Therefore, if dehydration takes place, the color of fish fillets will alter over time in storage because of the release or oxidation of pigments, leading to darkening or fading [162].

### 5.2. pH Stability

The rise in muscle pH during frozen storage is a result of the enzymatic breakdown of muscles and the creation of volatile basic compounds. Coatings have the ability to reduce the pH, thereby preventing the activity of endogenous proteases linked to lactic acid production via anaerobic glycolysis and reducing the release of inorganic phosphate, a by-product of ATP breakdown [30]. Jia et al. [163] found that during storage, because of fish degradation, ATP degradation products, inosine 5′-monophosphate (IMP) and hypoxanthine (Hx), can be measured and follow a decreasing trend for IMP and increasing for Hx. Following the death of aquatic organisms, the conversion of ATP to IMP is presumed to be a totally autolytic process; thus, if coating treatment can inhibit the further degradation of IMP, then a better seafood flavor is maintained. The application of a chitosan coating resulted in a notable enhancement of the physiochemical characteristics of frozen smoked herring, particularly in terms of pH levels [141]. Coatings made from functional fish protein powder derived from *Equulites klunzingeri* contributed to the enhancement of rainbow trout fillet quality by reducing the pH, total volatile base nitrogen, and free fatty acids in the fish meat. This improvement was attributed to the coatings’ effective barrier properties against moisture transfer, oxygen absorption, and microbial proliferation [164].

### 5.3. Avoiding Oxidation

At temperatures lower than 0 °C, lipid oxidation becomes the primary organoleptic factor, playing a crucial role in the quality and shelf life of fish products. Therefore, an oxygen barrier is essential. Chitosan’s oxygen barrier property is believed to reduce oxygen diffusion and inhibit lipid oxidation, as proposed by Sathivel et al. [165] on chitosan-coated salmon fillets. Additionally, the presence of natural antioxidants is also appreciated together with preprocessing methods, which can enhance the effectiveness of freezing and frozen storage [166]. By inhibiting oxygen permeability, edible coatings caused a reduced rate of enzymatic and non-enzymatic reactions, thereby minimizing the environmental influences on the fish during storage [30]. A notable example is the combination of chitosan and citric acid or licorice extract, where both compounds boosted the antioxidant capabilities of chitosan. A significant antioxidant effect on ovate pompano fillets (*Trachinotus ovatus*) stored at −18 °C for 6 months was demonstrated. In addition, coatings were able to reduce drip loss and prevent primary and secondary lipid oxidation, as evidenced by lower peroxide (PV) and thiobarbituric acid reactive substance (TBARS) values compared to the control group [144].

As mentioned earlier, antioxidants are substances that protect cells from oxidative processes through various mechanisms. The stability of antioxidants, particularly polyphenols, is influenced by factors such as pH, metal ions, exposure to light, temperature, oxygen, and enzymatic activity. The effect of cold and frozen storage on the antioxidant’s activity and stability depends on a series of intrinsic factors (e.g., composition and structure), while the role of extrinsic processing-related factors, such as freezing and storage temperatures, is ambiguous. In particular, many conflicting results are reported in the literature with high variability depending on the method of analysis used for antioxidant evaluation and data expression (fresh or dry weight). Other intrinsic raw polymer-coating properties (e.g., structure, ionic strength, viscosity), which in most studies are scarcely reported, contribute to the aforementioned discrepancies. Generally, the low temperatures used in freezing can reduce the kinetic energy of reactants. However, their effects on oxidation reactions can be complex. At low temperatures, increased oxygen solubility, the concentration of reactants in the non-frozen phase, and the crystallization of amorphous solutes can all promote chemical and enzymatic oxidation reactions. This complexity must be taken into account when considering the long-term storage of natural antioxidants for fish preservation [167]. For example, even at lower temperatures, the degradation of anthocyanins is still a concern. Most applications seek various methods to stabilize natural compounds due to the need for temperature stability or slow, controlled-release systems. For instance, when bioactive compounds, including antioxidants, are encapsulated—whether within a polymer coating matrix or different carriers—their stability improves.

Finally, due to the limited number of studies reported in the literature and the high variability in all parameters (the nature of coatings, antioxidants, mechanisms of action, and processing conditions), the effect of cold and frozen storage on the antioxidant’s activity and stability remains unclear.

### 5.4. Biopolymer Glazing

In contrast with traditional frozen storage methods that may result in a gradual decline in the quality of fish, the practice of frozen fish glazing with a layer of ice can help preserve its intrinsic [168] and sensory characteristics [169]. This glazing technique, particularly when incorporating hydrocolloids, acts as a protective barrier against quality deterioration and is comparable to edible coatings. Recent research has shown a growing interest in using edible polysaccharides [170] and functional antimicrobial and antioxidant polymers for ice glazing of fish [171].

Polysaccharides, for example, possess potent anti-freezing properties that enable them to effectively counteract alterations in fish protein texture, water loss, and migration. A consistent and well-applied layer on the fish’s surface is vital to minimize quality degradation resulting from exposure to factors like freezing and thawing rates, temperature fluctuations, storage conditions, transportation, distribution, and consumption temperatures. Conversely, an inadequate layer may compromise fish quality due to partial thawing and slow refreezing in cold storage [169]. The application of a glaze or coating solution is influenced by factors such as the application time, solution temperature, fish temperature, and the size and shape of the product. Lowering the temperature will result in a thicker coating. Edible coatings for frozen storage can be applied before freezing or on samples that are already frozen to imitate the glazing effect. Soares et al. [172] conducted a study on the impact of various factors, such as fish temperature, coating temperature, and dipping time, on the thickness of chitosan coating. Their findings indicated that decreasing the temperatures of the chitosan coating resulted in a greater coating thickness in the final salmon product [172]. For instance, a temperature drop of the coating solution from 8 to 2.5 °C at −25 °C led to an 80% increase in coating thickness. Moreover, dipping time was found to have implications for the safety aspects of the water glazing process/chitosan coating.

Xiao et al. [173] showed that the physical properties of polysaccharide-infused ice were affected by both the polysaccharide type and concentration. Increasing the polysaccharide concentration reduced the water activity in the supercooled polysaccharide solution. Furthermore, ice resulting from the solution exhibited smaller crystals, weaker compressive strength, slower sublimation rate, and reduced oxygen permeability. The effectiveness of the coating decreased in the following order: pullulan > gellan gum > k carrageenan > CMC > xanthan gum> and konjac glucommanan.

The same authors developed a unique water gradient edible film using konjac glucomannan and highly acrylic gum, with controlled swelling, which effectively combines the benefits of ice glazing and edible film properties [173,174]. By controlling the swelling, this innovative approach overcomes the limitations of both techniques. When frozen, the film’s higher water content portion acts as an ice glazing layer, while the remaining solid film maintains its function as a water and oxygen barrier.

Summary of recent literature studies on various coatings applied for fish preservation during frozen storage is given in Table 2.

## 6. Synergistic Effect of Edible Coating and Hurdle Technologies for Fish Preservation

Even though the effectiveness of edible coatings in preserving fish is undeniable, as demonstrated in the previous sections, they cannot be the sole packaging used for distribution and retail. As a result, efforts are being made to combine this method with other hurdle technologies like vacuum packaging (VP), modified atmosphere packaging (MAP), and cold plasma (CP). A promising alternative for improving the microbial quality of fish products stored at refrigeration temperatures has been demonstrated to be combined strategies. In order to achieve multitarget and reliable microbial spoilage control, hurdle technology makes use of existing or new distinct preservation techniques (also known as hurdles) that are effectively combined, whereas bacteria are being put under stress by the hurdles [182].

### 6.1. Synergies with Modified Atmosphere Packaging

A summary of some recent literature data is given in Table 3. Generally, MAP is known as a fast and cost-effective technology that inhibits spoilage microflora with substantial industrial potential for easy implementation. The gas in the package slows down the metabolic process of microorganisms, preventing them from growing and developing. This technique places the microorganisms in a largely dormant or semi-dormant state, thereby facilitating the longevity of packaged food. The packaging’s low concentration of oxygen prevents the auto-oxidation of lipids, while enzymatic hydrolysis is prevented by the packaging’s higher concentration of carbon dioxide [183].

### 6.2. Vacuum Packaging

Vacuum packaging presents the removal of air from the package and necessitates the application of a hermetic seal. In fish processing, VP can enhance ice storage or refrigeration shelf-life by postponing spoilage and decreasing the growth of aerobic spoilage microorganisms, *Enterobacteriaceae*, H_2_S-producing bacteria, and the total psychrophilic microflora [188]. Wholesale fresh fish meat is increasingly being packaged under a vacuum because it prevents shrinkage; preserves color; delays oxidation; and reduces thiobarbituric acid reactive substances, peroxide value, total volatile basic nitrogen, and pH value due to a low-O_2_ environment [188,189]. The addition of essential oil constituents, active antioxidants, and antimicrobials may improve sensory properties by providing a pleasant aroma, as demonstrated by sensory assessors prioritizing lemon verbena during VP fish storage [188].

Recently, authors [190] investigated whether refrigerated shrimp’s (*Penaeus indicus*) shelf life could be extended using a combination of vacuum and an edible coating made of basil seed gum and lemon essential oil. The research showed that coated and vacuum-packed shrimps had lower TBARS values compared to non-treated samples at the end of the 20-day refrigerated storage. Moreover, treated samples had improved microbial quality and a significantly reduced lactic acid bacteria (LAB) count on day 20 (*p* <  0.05), with acceptable color and odor. The shelf-life extension of shrimps was 16–24 days.

A synergistic effect of brown seaweed coating (at a concentration of 2%) in combination with VP showed the enhancement of antioxidant, biochemical, antimicrobial, and sensory qualities of reef cod (*Epinephelus diacanthus*) fillets stored under refrigeration with air-packed samples [191].

Other synergistic vacuum/coating effects were also shown, such as the extended shelf life of rainbow trout in cold storage with chitosan enriched with lemon verbena and zein enriched with sour orange peel [188,192]. This was also true for chitosan microparticles used to preserve gilthead sea bream fillets [105], pectin–plant essential oil used to preserve large yellow croacker (*Larimichthys polyactis*) [193], and lactoperoxidase system–whey protein used to preserve pike–perch fillets (*Sander lucioperca*) [194].

## 7. Use of Edible Coatings for Ready-to-Eat Fish

Consumers increasingly favor ready-to-eat (RTE) fish due to its convenience, health benefits, nutritional value, mild preservation, extended shelf life, and appealing quality. As the global population grows and ages, RTE fish becomes an even more valuable source of high-quality nutrition. RTE fish and foods are fully cooked or prepared, allowing for immediate consumption without the need for further cooking or processing to eliminate harmful microorganisms.

The shelf life of chilled RTE items is short, necessitating proper handling to maintain quality and safety. The utilization of coatings for RTE seafood products remains somewhat limited despite the numerous potential benefits they offer [24,195]. Existing research is described below.

Bremenkamp and Souza-Gallagher [24] developed an edible coating from maritime- sources, based on chitosan and alginate, to control the degradation of RTE baked fish fillet. Material properties, coating composition, and the development process were optimized. The authors showed that 1% (*w*/*v*) chitosan (with plasticizer) or 1% (*w*/*v*) alginate coating (without plasticizers or crosslinkers) were the best in controlling the quality parameters and maintaining product safety. The shelf life of chitosan-coated samples was three times longer than that of the uncoated ones when kept at 4 °C. Moreover, the tested coatings were shown to be efficient preservation tools even under abusive storage conditions.

According to another study, chitosan coating improved barracuda (*Sphyranea sphyranea*) fish sausage quality when it was kept cold (4 °C) for 12 days. The results indicated that coatings significantly impacted microbial stability, texture, and sensory quality [196]. Furthermore, in another study, chitosan coatings enriched with peppermint essential oil emulsion prevented microbial growth in fish meatballs during cold storage (4 ± 1 °C) [197].

Fishbone gelatin from giant snakehead (*Channa micropeltes*) at 2% improved the water holding capacity and led to an acceptable sensory score in frozen shrimp [198].

Rainbow trout were found to have a longer shelf life when coated with zein coatings enriched with essential oil and the extract of *Pimpinella affinis* (both at 1 and 2%). Compared to controls, total volatile basic nitrogen and pH parameters in coated fish samples decreased, suggesting lower oxidation. Bacterial counts in coated samples also continued to be below the acceptable level, and treatments containing *Pimpinella affinis* had better sensory scores compared to control treatment during storage [199].

## 8. Factors Affecting Coating Process of Fish

Choosing an appropriate coating method is crucial as it significantly affects both the preservation efficacy of the coating on food products and the overall production costs and efficiency. Although coating fish offers several benefits, challenges remain that hinder its widespread commercialization. To better address the practical applications and deposition techniques of edible coatings and films for fish, further scientific and applied research is essential. Currently, the most common coating methods include dipping, spraying, fluidized bed, and panning. Dipping is straightforward and often preferred in laboratory settings due to its simplicity. However, in industrial applications, dipping can lead to issues such as dilution and contamination. Conversely, spraying and brushing do not have these drawbacks and provide a more uniform coating, better thickness control, and multiple-layer applications. However, they require more complex equipment [200] but are still preferable on a commercial scale.

Despite their numerous advantages, edible coatings face several limitations and challenges that limit their application on an industrial scale:

- For coatings enriched with essential oils having a sharp, characteristic aroma and flavor [201], the appropriate selection of a suitable polymer, optimized concentration and viscosity, flavor enhancers, and encapsulation tools can be used to improve the aroma barrier and retention properties so as to minimize some of the mentioned limitations.
- Poor film-forming properties, poor adhesion properties, and instability may limit their commercial applications.
- Coatings may not affect or alter, or as little as possible, the fish’s aspect (color, shininess, etc.).
- A lack of materials with the required functionalities (a poor moisture barrier due to the hydrophilic nature, poor temperature, and relative humidity control).
- High investment costs for coating equipment installation.
- Regulation- and safety-related issues, since there is no recommended standard describing the applications for different edible coatings.

## 9. Adhesivity Issues

The efficiency of edible coatings is heavily dependent on the compatibility between fish surface and coating wettability, which also impacts coating thickness and uniformity [202]. The formulation is fundamental in the coating design, influencing the effective spreading, which is ideally even on the fish surface. During the coating process, the product is wet, the solution may penetrate the product, and then both may adhere to each other. Consequently, this process also influences the permeability and color properties of the coatings after drying. The coating should be easily applicable, have good adhesive characteristics, and dry quickly with uniform thickness. Furthermore, the coating’s performance and structural integrity must be maintained during long-term storage, especially if frozen conditions are applied. Coatings must be flexible enough to adapt to morphological changes, such as desiccation and mechanical damage (possibly occurring during handling).

Three parameters need to be considered: (a) surface free energy, (b) surface wettability, and (c) coating wettability.

(a) Surface free energy (SFE) determines the adhesive properties of a coating formulation. Food surfaces with low SFE (<75 mN m^−1^) are called “low energy” or hydrophilic surfaces [203]. The authors found that the surface tension of the shrimp fillets was 59.93, indicating a low-energy surface with dispersive and polar components of 14.14 and 45.79 mN m^−1^, respectively [204]. The critical surface tension is determined using the Zisman method [205]. The principle of this method says that the contact angles formed by a liquid (coating) on low-energy surfaces is a linear function of the surface tension of that liquid (coating) [206]. For hydrophilic surfaces such as fish, the polar component should have a positive value. The dispersive component is associated with the non-polar components, like lipid fractions, that result in its lower values.
(b) A product’s wettability is primarily determined by the chemical composition and the microscopic geometry of its surface, both of which can be easily affected by texture, physical roughness, surface microstructure, and chemical heterogeneity. The contact angle of the food substrate will also provide information regarding the formulation that will be most effective at coating the substrate. A lower contact angle indicates better wetting.
(c) The coating wettability is described by adhesive forces, which promote the spreading of the coating on a fish surface, and cohesive forces, which promote the contraction of polymer chains of the coating solution. Adhesive and cohesive forces are used to calculate the spreading coefficient. Consequently, wetting behaviors mainly depend on the balance between these two forces [207,208,209,210].

There is no proposed optimal coating formulation, and if possible, adhesive and cohesive forces should be measured for specific applications. For example, de Lima Silva et al. [206] showed that increasing the carrageen concentration had a negative effect on the adhesion coefficient on meat, while others [207] showed that the chitosan concentration had a positive effect on the adhesion coefficient but a negative effect on the spreading coefficient in *Scomberomorus brasiliensis* fish fillets. A higher viscosity can assure a superior adhesion since it has a greater resistance to the movement. Contrarily, for gelatin coatings, increasing the biopolymer concentration decreased both the adhesion and spreading coefficients on Nile tilapia (*Oreochromis niloticus*) fish fillets [210].

The cohesion coefficient can be reduced using plasticizers [206]. Indeed, glycerol increased the spreading coefficient of chitosan coating, resulting in its improved adhesion compared to *Scomberomorus brasiliensis* fillets [208]. In this example, the concentration of plasticizer did not have any impact. In another study, as the concentration of the plasticizer increased, the cohesion coefficient of chitosan coatings decreased, while the opposite was observed for gelatin coatings. The optimized wettability on shrimp (characterized as low energy surface) was achieved for coatings with 1.0% chitosan and 0% glycerol (with spreading coefficient (W_s_) of = −12.934 mN m^−1^) and 1.0% gelatin and 0% glycerol (W_s_  =  −5.588 mN m^−1^) [206]. In the case of carrageenan, at low polymer concentrations, glycerol did not impact cohesion, while when used at higher concentrations in a study performed on chicken meat, glycerol was shown to have the opposite effect. Therefore, the critical polymer concentration was determined to be <1% [206].

## 10. Conclusions

Edible coatings for fish preservation play a vital role in extending shelf life and maintaining freshness and fish qualities. These coatings create a protective barrier, reducing moisture loss, lipid oxidation, and microbial growth. The effectiveness of these coatings depends heavily on the storage temperature, as lower temperatures (such as refrigeration or freezing) enhance their ability to preserve fish quality by slowing down biochemical reactions. Changes in temperature slow down the degradation processes in fish flesh. However, changes in cold-stored and frozen fish are quite different. While lowering the temperature to 4 °C slows down reactions, there is still a risk of autolysis, oxidative stress, and microbial contamination. Therefore, the principal aim of a coating is to stop oxidation since it will also lead to autolysis and spoilage. Oxygen barrier coatings should therefore be used with even more pronounced ceasing if enriching with antioxidants that will capture oxygen from the external atmosphere but also of pro-oxidants in fish flesh present after rigor mortis. When temperatures decrease below 0 °C, it inhibits biological and chemical reactions and minimizes the enzymatic activity. However, water in fish flesh changes to ice crystals that can lead to freezer burn, dehydration of the surface, and oxidation. Thus, the primary objective of an edible coating during frozen storage is to offer sufficient barriers against water vapor and oxygen influence while preserving the desired pH level. These coatings should then preferably be more hydrophobic than hydrophilic since water portions in the coating itself might change the coating structure during freezing by breaking down a coherent polymer matrix. The composition of the coating (e.g., polysaccharides, proteins, and lipids) must meet product requirements, providing an appropriate balance between organoleptic and hygienic properties and protection. Incorporating antioxidants (such as natural extracts or essential oils) further enhances coating performance by neutralizing free radicals and preventing oxidation. Efficient coatings need to withstand varying storage temperatures and packaging processes (MAP, vacuum, etc.) while ensuring consistent antioxidant or antimicrobial release. Optimal formulations maintain the fish’s texture, color, and nutritional value, addressing both preservation and consumer safety. The best way to combine naturalness and thus consumer acceptance for the coupling of fish and an edible coating is to use additives and ingredients that are mainly extracts from the seafood industry.

## Figures and Tables

**Table 1:** Antimicrobial and antioxidant activities of various edible coatings designed to reduce fish-specific microorganisms.

Microorganism	Films/ Coating	Active Molecule	Bioactivity		Fish Type	Impact on Product	Ref.
			AM	AO			
Aerobic plate count	CS	2%, 3%, and 4% (*w*/*v*) CS	X	X	Smoked herring fish	More than 4 log_10_ CFU/g reduction in aerobic plate count when sample was coated by chitosan at 4%. CS coating (2%, 3%, and 4%) significantly increased TPC, total flavonoid contents, and DPPH% activity (>80% of AO) of coated fish	[141]
Total viable organisms	GEL/ CS (3:1) 8% (*w*/*v*)	Clove EO/7.5% (*v*/*w*)	X		Salmon fillets	Increase in shelf life up to 11 days (control sample 4 days) with 4.5% (*v*/*v*) clove	[142]
Total viable organisms	1.5% CS	GSE0.2% (*w*/*v*) TP/0.2% (*w*/*v*)	X		Red drum fillets	Increase in shelf life up to 16 days with 0.2% (*w*/*v*) GSE or TP	[143]
Total viable organisms	1.5% (*w*/*v*) CS	CA0.5% (*w*/*v*) Licorice ex.1% (*w*/*v*)	X		Japanese seabass fillets	Increase in shelf life up to 12 days when 0.5% (*w*/*v*) CA in CS	[144]
Total viable organisms	Furcellaran and GEL	Thyme or rosemary (ROS)	X		Carp fillets	Coatings with ROS extract showed the highest efficacy: 30% decrease in total viable count after 6 days of storage	[145]
Total viable organisms	Xanthan gum	Daphne and basil extract	X		Rainbow trout	TVC of fish with active coating was significantly improved: 5.3 for daphne and 5.51 log CFU g^−1^ for basil (control of 7.95 log CFU g^−1^)	[146]
Total mesophilic bacteria	CS	*Artemisia* *Dracunculus* EO	X		*Scomberoides commersonnianus*	After 16 days of storage, total mesophilic bacteria count reduced by 1.5 log CFU/g with active coating	[147]
Total mesophilic bacteria	CS	*Zataria* *multiflora* EO	X		Asian sea bass (*Lates calcarifer*)	After 16 days of storage period, a 1.8 log CFU/g reduction for total mesophilic bacteria with active coating was noted	[148]
*E. coli*	CS, EDTA	Nisin	X		Fresh grouper fish fillet	CS/nisin/EDTA films reduced *E. coli* by 1.4 and log CFU/cm^2^ with activity	[149]
*E. coli*	GEL	TEMPO-oxidized cellulose nanofibers (TCNFs) and CAR (100, 300, and 500 mg)	X	X	Fish fillets	Diameter of inhibition area on potato dextrose agar increase until 7, 16, and 19 mm after CAR addition at 100, 300, and 500 mg, respectively. When CAR content reached 500 mg, DPPH· and BTS^+^ scavenging rate of the film were 37% and 46%, respectively	[109]
*L. monocytogenes*	CS	Nisin, Na-LAC, Na-ACT, PS, Na-BEN	X		Cold-smoked salmon	Active coating completely inhibits the growth of *L. monocytogenes* for at least 6 weeks compared to uncoated sample	[150]
*L. monocytogenes*	GEL	Nisin, thymol	X		Rainbow trout	*L. monocytogenes* reduced by 0.65 log CFU/g with active coating	[151]
*Pseudomonas* spp.	ALG	Thyme, oregano, and pimento (0.5, 1, 1.5% (*w*/*w*)	X		Carp fillets	Coatings showed a reduction in *Pseudomonas* from 7.35 for uncoated sample to 3.49 log CFUg^−1^ for coated ALG/oregano (1.5% (*w*/*w*)) after 6 d	[48]
*Pseudomonas*	CS	0.25 and 0.5% (*w*/*v*) lemon or thyme EO	X	X	Grass carp fillets	Active coatings showed a reduction in *Pseudomonas* from 7.45 (uncoated sample) to 5.46–5.67 log CFU/g (active with lemon or thyme EO at 0.5% (*w*/*v*). Active coatings delayed increase in TBARs by 33%	[85]
*Pseudomonas*	GEL	TEMPO-oxidized cellulose nanofibers and CAR 100, 300, and 500 mg	X		Fish fillets	Active gelatin coating with TEMPO-oxidized cellulose nanofibers and 500 mg of carvacrol showed a reduction in *Pseudomonas* from 5.5 to 3.6 log CFU/g after 6 days of storage	[109]
*Staphylococcus aureus*	Basil and *Lepidium perfoliatum* gum	*Foeniculum vulgare* EO (2%)	X		*Oncorhynchus mykiss* fish fillets	After 28 days of storage, log reduction for *Staphylococcus aureus* was 3.59 log CFU/g for active coated sample compared to uncoated sample	[152]

AO—antioxidant activity; AM—antimicrobial activity; ALG—alginate; GEL—gelatin; CS—chitosan; SOR—sorbitol; GLY—glycerol; EO—essential oil; GSE—grape seed extract; TPs—tea polyohenols; CA—citric acid; EDTA—ethylenediamine tetra acetic acid; CFU—colony-forming unit; TVC—total viable counts; Na-LAC—sodium lactate; Na-ACT—sodium diacetate; PS—potassium sorbate; Na-BEN—sodium benzoate; CAR—carvacrol.

**Table 2:** Recent literature studies on various coatings applied for fish preservation during frozen storage.

Coating Type	Fish Species	Combined Processing (e.g., Active Compound)	Temperature and Storage Time	Effect on Quality				
				Physico-Chemical Quality	Microbial	Sensory Quality	Additional Functionality	Reference
Chitosan	Atlantic salmon (*Salmo salar*)	Water glazing and chitosan coatings	−22 °C, 9 months	No impact on weight and coating loss, significant pH value drop	Well-controlled	++ impact on color but no significant effect on K-value		[172]
	Smoked Herring (*Clupea* *harengus*)	/	−18 °C, 3 months	Improved, including pH, TVB-N, trimethylamine, TBARS, free fatty acid %, acid number	↘↘ aerobic bacteria, complete suppression of psychrotrophic, *Enterobacteriaceae*, yeast, and mold	Improved sensory attributes	Improved phenolic and flavonoids, strong antioxidant activities	[141]
	Rainbow trout (*Oncorhynchus mykiss*)	Pomegranate peel extract	−18 °C, 6 months	Improved. ↘↘ pH, TBARS, TVB-N, protein solubility, no decrease in sulfhydryl groups	Improved, lower values of LAB and molds	Improved texture, but negative impact on the color		[175]
	Tambaqui (*Colossoma macropomum*)	Clove essential oil	−18 °C, 120 days	Delayed lipid oxidation, reduced pH and moisture	Inhibited psychrotrophic bacteria	Negative impact on sensory evaluation	Antioxidant activity	[31]
	Channel catfish (*Ictalurus punctatus*)	Acetic and aspartic acid	−20 °C, 180 days	Lipid oxidation controlled, reduced drip loss and cooking loss	Inhibited growth of aerobic mesophilic and psychrophilic bacteria	Good color and texture; preferable use of formulation with non-pungent aspartic acid		[176]
	Ovate pompano (*Trachinotus ovatus* L.)	Citric acid or licorice extract	−18 °C, 6 months	Inhibition of primary and secondary lipid oxidation (PV, TBARS), decreased drip loss		Enhanced antioxidant effect		[144]
	Rohu (*Labeo rohita*)	/	−18 °C, 14 weeks	pH, TBARS, and K-value of 1% and 2% CS-treated fish fillets were acceptable up to 14th week of storage, while TVB-N value was permissible up to 12th week	Improved microbial stability	Acceptable sensory attributes up to 12th week, compared to control fillets (unacceptable after the 6th week)		[177]
Sodium alginate	Rainbow trout (*Oncorhynchus mykiss*)	EtOH *Prosopis farcta* extract (0.5%) and curcumin nanoparticle	−18 °C, 6 months	Stable chemical properties	Good microbial stability		Strong antioxidant activity	[178]
Water extract	Nile tilapia (*Oreochromis niloticus*)	Green tea or olive leaf extract	−8 °C, 105 days	Reduced fat content	Improved microbial stability	Lower total quality index		[42]
Corn-zein	Hybrid Striped Bass	Nisin or lemongrass EO		(*Morone chrysops* × *Morone saxatilis*)	Strong antibacterial effect			[179]
Combined coatings								
Persian gum/chitosan	Silver carp (*Hypophthalmichthys molitrix*)	Garlic (*Allium sativum* L.)	−18 °C, 6 months	Significantly reduced lipid oxidation		Good sensory scores	Good antioxidant activities	[180]
CS, CS/GEL; WPI/XG	Tilapia (*Oreochromis niloticus*)		−18 °C, 12 months	Improved	Improved	Sensory score better in coated samples	Chitosan had best preservation effect	[181]

TBARS—thiobarbituric acid reactive substances; TVB-N—total volatile basic nitrogen; LAB—lactic acid bacteria; PV—peroxide value; CS—chitosan; GEL—gelatin; WPI—whey protein isolate; XG—xanthan gum; EO—essential oil; ↘↘ means significant reduction; ++ means very positive.

**Table 3:** Use of MAP in combination with edible coatings and other hurdle technology for fish preservation.

MAP Cond. (CO_2_/O_2_/N_2_) (%)	Coating Type	Fish	Effect	Storage Type	Ref.
50/5/45	WPI enriched with oregano and thyme essential oil (WPI-EO)	Hake fillets (*Merluccius merluccius*)	Synergistic effect WPI-EO and MAP - Significant extension of the lag phase; reduction in total viable counts and H_2_S-producing bacteria	COLD: 4 °C, 16 days in high-barrier E/PP/EVOH/PP film	[182]
100% air, 40%, or 70% CO_2_; N_2_ balance	Chitosan	Maki sushi	- Increased microbiological stability; - Color properties preserved in low-CO_2_ MAP	COLD: 4 °C and 8 °C, 8 days	[184]
60/5/35	LBG/sodium alginate enriched with daphnetin emulsions (0.16, 0.32, and 0.64 g L^−1^)	Turbot (*Scophthalmus maximus*)	- Water release from fish muscle fiber was either delayed or converted into free water based on muscle fiber destruction, maintaining the excellent fish quality	COLD: 4 °C, 18 days, PE bags	[93]
40/0/60 40/30/30 60/10/30 60/0/40	Gelatin coating enriched in eugenol	Sea bass (*Lateolabrax maculatus*)	- In 60%CO_2_/10%O_2_/30%N_2_ packages, the best quality was achieved - Presence of active gelatin coating promoted the shelf-life and fish safety due to its antimicrobial activity and resistance to oxidation	SUPERCHILLING: −0.9 °C, 36 days	[185]
80/10/10	Chitosan-based composite coating with ε-polylysine	Pompano (*Trachinotus ovatus*)	- Reduced total bacterial count and total psychrotrophic bacteria count - Improved color - Lower levels of TBARS and TVB-N -Storage life extended without influencing sensory quality	COLD: 4 °C, 14 days	[186]
60/0/40	CS-flaxseed mucilage films with *Ziziphora clinopodioides* EO (0.25%, 0.5%) and sesame oil (0.75%)	Minced trout fillets (*Scophthalmus maximus*)	- Improved storage stability of samples packed in MAP - Shelf-life: MAP > VP > aerobic - Better quality parameters and oxidation stability in coated samples	COLD 4 °C, 16 days	[187]
Combined with Cold plasma (CP)	Chitosan enriched with wampee seed EO (CS-WEO)	Golden pompano (*Trachinotus blochii*)	- CP had antibacterial effect - CS-WEO film could inhibit microbial proliferation, maintain fillet texture profiles, and inhibit lipid hydrolase - Enhanced preservation	COLD: 4 °C, 14 days	[29]

WPI—whey protein isolate; LBG—locust beam gum; CS—chitosan; VP—vacuum packaging; EO—essential oil; CP—cold plasma.
